# A Survey of Italian Physicians' Opinion about Stem Cells Research: What Doctors Prefer and What the Law Requires

**DOI:** 10.1155/2014/480304

**Published:** 2014-04-30

**Authors:** Paola Frati, Matteo Gulino, Arianna Pacchiarotti, Stefano D'Errico, Lorella Sicuro, Vittorio Fineschi

**Affiliations:** ^1^Department of Anatomical, Histological, Forensic and Orthopaedic Sciences, University of Rome La Sapienza, Viale Regina Elena 336, 00161 Rome, Italy; ^2^Istituto Neurologico Mediterraneo, Neuromed, IRCCS, 86170 Isernia, Italy; ^3^Department of Obstetric Gynaecological and Urological Sciences, “Sapienza” University of Rome, 00161 Rome, Italy; ^4^Legal Medicine Unit, Hospital “Campo di Marte”, 55100 Lucca, Italy; ^5^Italian National Institute of Statistics, 34 Via Caduta del Forte, 65121 Pescara, Italy

## Abstract

To evaluate the Italian physicians' knowledge/information level about the therapeutic potential of stem cells, the research choice between embryonic and cordonal stem cells, and the preference between autologous and heterologous storage of cordonal stem cells, we performed a national survey. The questionnaire—distributed to 3361 physicians—involved physicians of different religious orientations and of different medical specialities. Most of the physicians involved (67%) were Catholics, and the majority were gynaecologists and paediatricians (43%) who are mainly in charge to inform future mothers about the possibility of cordonal stem cells conservation. The majority of the physicians interviewed do not have specific knowledge about stem cells (59%), most of them having only generic information (92%). The largest part of physicians prefer to use umbilical cord blood cells rather than embryonic stem cells. Nevertheless, a large percentage of physicians were in favour of embryo research, especially when embryos are supernumerary (44% versus 34%). Eighty-seven % of the physicians interviewed proved to have a general knowledge about stem cells and believe in their therapeutic potential. They prefer research on cordonal stem cells rather than on embryo stem cells. Although they are in favour of heterologous stem cells donation, they still prefer cryopreservation for personal use.

## 1. Introduction

Stem cells research is recently reproposing the paradigmatic case of the reciprocal influence between science and ideological (e.g., political, religious, economic, and social) issues. More specifically, an important question deals with the impact upon national regulation and social behaviours of the scientific community experts' advice regarding stem cells research and its exploitation for health care purposes. Stem cell-based therapies, exploited in different clinical scenarios, have been recently the key matter of an important debate in the Italian as well as in the international scientific community due to the case of Stamina Foundation. Translational medicine thus represents a stem of scientific research that aims to move “from bench to bedside” or from laboratory experiments through clinical trials to point-of-care patient applications. The “prevalent theme” of this stem of research is without doubt represented by the regenerative medicine, with all the stem cell-based approaches corresponding to the zenith of the epiphenomena [1].

This represents one of the most recent examples of the conflict which may occur between science and political and sociocultural issues [2]. Indeed, while on one hand science and the diffusion of scientific education are widely believed to foster democratic values and maintain political [3], moral, and economic stability of modern democracies, on the other hand political prejudices together with religious and other ideological interests as well as economic issues may limit and quench the advancement and diffusion of scientific research and with its potential applications [4]. This mainly occurs in scientific fields which are likely to deeply impact the evolution of ideologies and behaviours (i.e., politics) of a country, which in some conservative way seems to protect itself from historical changes.

To this regard, a scientific, ethical, and political debate is recently reviving in Italy about the utilization of stem cells in regenerative medicine. In this debate, considerable differences of opinion exist depending on the derivation of the stem cells themselves: “adult” stem cells which are found in a variety of tissues in the foetus and after birth (more specialized “multipotent” cells with an important function in tissue replacement and repair) and embryonic stem cells (hESC) which can only be derived from preimplantation embryos (“pluripotent” cells which have the ability to form cells of all tissues of the adult organism and could be used to develop cell-based therapies) [5].

The lawfulness of the research concerning the clinical employment of stem cells derived from adult tissues or from the umbilical cord (either from spontaneously or voluntarily aborted foetuses) would not seem to raise insurmountable questions. However, the question about the use of hESC appears charged with deeper ethical tensions.

The debate about human preimplantation embryos for research on ESC and using of umbilical cord blood stem cells has been reported to involve scientists, including the expert ones actively operating in this specific field of research. Poor information has been however reported on the attitudes of medical professionals (e.g., physicians) who are closer to patients or to the public opinion than specialized research scientists.

Fast forward to current evidence-based practice concerning translational medicine applied to organ's regeneration and exploited by different stem cell, our analysis can provide better insights into the more direct impact that these issues have on professional choices or into their influence on the patients' choice (e.g., about pursuing the alternative of nationally permitted therapeutic strategies or choosing more permissive countries to afford suitable stem cell therapies).

To this aim, we investigated the opinion of 3361 Italian physicians about the use of human embryos generation for isolation of hESC, about the stem cells clinical potential, and about related ethical and juridical issues.

## 2. Materials and Methods

The survey, self-administered and anonymous, was performed through an e-mail based questionnaire sent to a sample of 3361 physicians, males and females, aged from 30 to 70 years (Table 1).

The physicians involved worked in all areas of medicine (gynaecology and obstetrics, psychiatry, surgery, forensic medicine, paediatrics, dentistry, neonatology, and anaesthesiology). Physicians who do not normally deal with stem cell-based treatments have been surveyed. Special attention was given to gynaecologists and paediatricians, since they are in charge of the information delivered to future mothers about the possibility of cryopreserving their own son stem cells (Table 2).

Specifically, the questionnaire develops 14 points concerning the following areas: (i) knowledge and opportunities of human embryonic stem cells research; (ii) attitudes/opinions about hESC research; (iii) umbilical cord blood cells explant and storage techniques; (iv) differences between heterologous and autologous storage; (v) opportunity to donate surplus embryos actually stored for research purposes (Table 3).

Descriptive and inferential statistical data was performed. In particular, both the frequency and percentage of each type of reply for the different categories were calculated. A bivariate evaluation was then carried out in order to analyse the relationship between pairs of variables. The Chi square test was especially used for investigating significant statistical differences between categorical variables; a *P* value of less than 0.05 indicated statistical significance.

In particular, we have analysed the relationship between religion and the responses to the questionnaire, including medical area of belonging and answers, and also between certain pairs of responses thought to be more interesting to compare.

## 3. Results

The questionnaire was distributed to 3361 physicians and 377 completed surveys were received. The response rate was 11% and 88% of the respondents were aged from 47 to 59 years. Forty-three % (*n* = 162) of the physicians involved worked in the field of gynaecology and obstetrics and 41% (*n* = 156) were paediatricians. The questionnaire involved physicians having different religious orientations and life attitudes (Catholics, Hebrews, Christians, atheists, and agnostics). Most of physicians involved were Catholics (56%; *n* = 210) while 9% (*n* = 35) were atheists/agnostics, 1% (*n* = 2) were Jews, 2% (*n* = 6) were of other religions, and 21% (*n* = 79) were unknown (Table 4). The high percentage of Catholics elucidated a preference for cordonal stem cells research rather than hESC research. These outcomes meet Pope Benedetto XVI's thinking who stated in this specific matter that “No ethical problem rises when stem cells are collected from nonembryonic tissues, cordonal blood at birth, or dead foetuses.” Nevertheless, a large part of physicians involved (44%; *n* = 165) were in favor of research on supernumerary embryos. Interestingly, among different medical areas, the responses to the questionnaire showed statistically significant differences (*P* = 0.001). With regard to the question “Have specific knowledge about stem cells,” the gynaecologists and obstetricians are well informed in a specific manner on the subject (43%), followed by the other medical areas' specialists (28%) and paediatricians (26%).

Even the participation in conferences or training courses is the prerogative of the gynaecologists (44% versus 20% of paediatricians and 20% of other medical areas).

The vast majority of gynaecologists also personally received information/communication methods on the harvesting procedure of stem cells from umbilical cord (78% gynaecologists versus 30% paediatricians and 22% of other physicians, *P* < 0.001). Again, gynaecologists are aware of the Italian legislation on the collection/storage of stem cells from umbilical cord (76% gynaecologists versus 42% paediatricians and 35% of other physicians, *P* < 0.001). Finally, gynaecologists are aware of the differences between autologous and heterologous stem cells from umbilical cord (81% gynaecologists versus 42% paediatricians and 41% of other physicians, *P* < 0.001).

For the other questions, there are no statistically significant differences in respect to the medical areas of specialty.

Regarding the relationship between religious belief and the answers to these specific questions, we found statistically significant differences with the question regarding the preference about the research/experimentation on human embryos. It is noticeable that doctors of other religions are more prone to this type of research (56%) than Roman Catholic doctors (31%). The relationship between religion and the question regarding the opinion on the research/experimentation on supernumerary human embryos is statistically significant (*P* < 0.001).

Only the 41% of catholic doctors agree with human embryo research with a high propensity towards the use of supernumerary human embryos.

For the other questions, there are no statistically significant differences regarding religious belief.

### 3.1. Knowledge of Stem Cells Research and Application

The analysis demonstrated that the majority of the physicians interviewed did not have specific knowledge on stem cells (59%; *n* = 223), most of them having only generic information (92%; *n* = 347). The largest part (65%; *n* = 248) of physicians involved did not attend vocational and training courses or meetings regarding stem cells, but were interested in such an issue (70%; *n* = 265), suggesting that they believe in the potential benefits of developing stem cells strategies.

We noted moreover that, among doctors who have specific knowledge about stem cells, 98% declared to be “aware of the possible therapeutic applications,” while among doctors who do not have specific knowledge about stem cells 90% are aware of the therapeutic potentiality (*P* = 0.005). Only 2% of the doctors who have specialized knowledge do not know the possible therapeutic applications.

Fifty-four % of physicians who “have specific knowledge about stem cells” participated in conferences/seminars/training courses with sessions on stem cells, while only 19% without specific knowledge about stem cells participated in conferences (*P* < 0.001).

We observed a statistically significant difference (*P* < 0.001) among the doctors who have participated in conferences/seminars/training courses and who have personally received information on methods for stem cells collection from umbilical cord. Again, seventy-eight % of those who participated in conferences received information on methods of sampling from the umbilical cord, while only 37% who received information on cord blood storage have not participated in conferences. Finally, we observed statistically significant differences (*P* < 0.001) between participants in conferences with awareness of the Italian legislation on the collection/storage of stem cells from umbilical cord versus nonparticipants. Seventy-nine % of doctors who participated in conferences are aware of the legislation, while 46% of doctors are informed about the Italian law despite not having participated in conferences or seminars or training courses.

### 3.2. Human Embryonic Stem Cell Research

**Figure 1:**
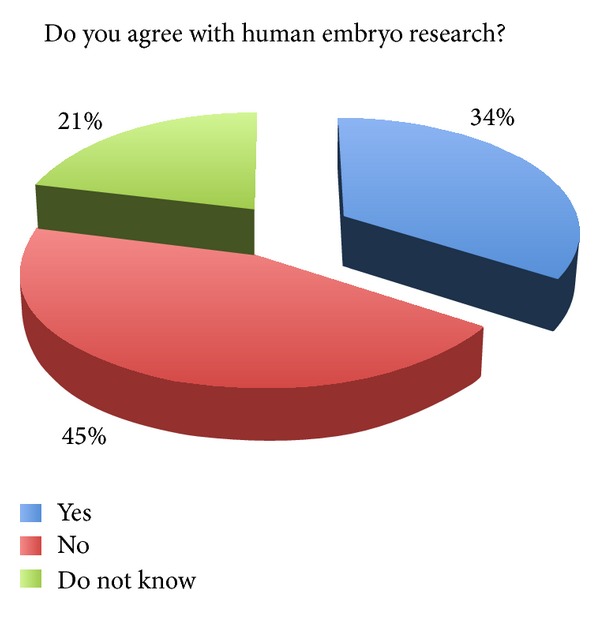
Human embryo research.

**Figure 2:**
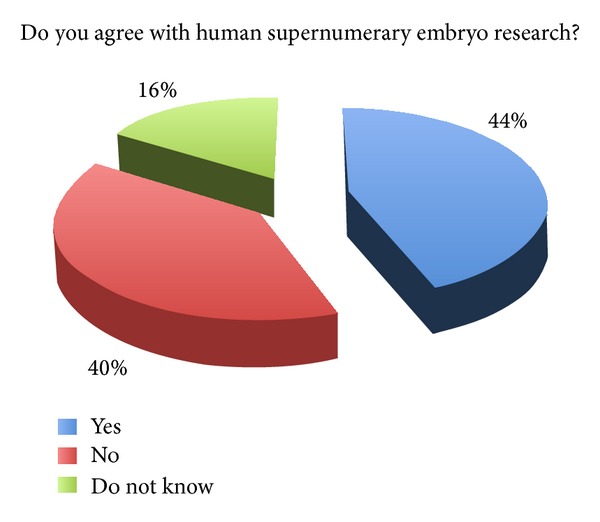
Human supernumerary embryo research.

The survey showed how embryonic stem cells research continues to be a tricky question. Accordingly, the majority of the physicians involved (87%; *n* = 330) wished to preserve the embryos' potential life. Indeed, a large part of the physicians involved preferred collecting umbilical cord stem cells rather than embryonic stem cells. The majority of them (45%; *n* = 173) did not approve the use of human embryos for research, while about one-third (34%; *n* = 126) answered in favour of it (Figure 1). With reference to the use of human supernumerary embryos for research purposes, the survey showed an increased percentage of favourable opinions (44% versus 34%) and the reduction of the percentage of unfavourable opinions (40% versus 45%) (Figure 2). These results show that the majority of physicians involved were not in favour of using human embryos for research, while they were in favour if such embryos are anyway destined to destruction or alternatively to be kept stored forever.

We observed statistically significant differences between the two questions related to the opinion more or less “conducive to research/experimentation on human embryos” and “supernumerary embryos” (*P* < 0.001). Ninety-six % of the doctors are in favour of both trials and 83% contrary to both, while almost sixteen % (16.7%) of physicians that approve research on the embryos do not agree on the testing of embryos and 4% who agree with the experiments of embryos are not for that of the supernumerary embryos.

### 3.3. Umbilical Cord Blood Cells Collection and Storage Techniques

Most of the physicians involved had only general information about stem cells supply. Ninety-two % of them only had knowledge of the possibility to isolate stem cells both from human embryos and from umbilical cord, lacking instead specific information about the techniques and the legislation.

Indeed, about a half (47%; *n* = 179) did not have specific information about stem cells collection from umbilical cord blood and 40% (*n* = 150) did not have knowledge about the Italian legislation on the matter. Not even many physicians were familiar with the difference between heterologous and autologous storage of stem cells from umbilical cord blood (54%; *n* = 203 positive answers compared with 46%; *n* = 174). The scientific misinformation about the difference between heterologous and autologous storage prevented physicians from considering the strictness of Italian legislation. Stem cells storage for public health purposes was accepted by 91% (*n* = 343) of physicians who were willing to donate umbilical cord stem cells of their child. About two-thirds (73%; *n* = 275) of the physicians involved wanted to preserve umbilical cord stem cells of their own child for personal use (Table 3).

## 4. Italian Legal Context

Italy has adopted very strict rules on the use of both human preimplantation embryos for research on ESC and umbilical cord blood stem cells, also expressly prohibiting the creation of human embryos for research or experimentation purposes. In Italy, the legal protection of the embryo is regulated since 2004 by the law on medical assisted reproduction (Law 40/2004), which, with a few concise and peremptory words, is to “liquidate” one of the emerging questions in scientific research. Such an extensive and complex subject—it is sufficient to stress once again the different implications of the various research technologies: cloning; adult stem cells; foetal stem cells; and embryonic stem cells—would require an appropriately complex law [6–8]. The law prohibits the creation of human embryos for research or experimentation purposes (art. 13). Manipulating embryos is allowed only if appropriate for therapeutic or diagnostic purposes and when no alternatives exist. However the Law n. 40/2004 does not state any rule regarding either the destiny of surplus embryos derived from in vitro fertilization (IVF) procedures, performed before the law itself was enforced, and actually stored in the various centers of assisted reproduction technologies (ART), or the potential research use of hESC imported from foreign countries. Moreover, the cryopreservation of umbilical cord stem cells is permitted exclusively for a public use and without remuneration [9]. The autologous storage for personal purposes is permitted only in case of serious diseases and in case of families with a high risk of genetically transmitted diseases.

The export of stem cells (even if they are harvested in Italy) for personal purposes is allowed at the expenses of the applicant after obtaining a ministerial authorization (art. 2, 9th paragraph, Ministerial Decree n. 42886/2009) [10]. Instead, the export of umbilical cord is strictly forbidden (art. 27, Decree n. 191/2007). Cord blood storage is allowed exclusively to public institutions (art. 1, Ministerial Decree n. 42886/2009).

## 5. Discussion

The aim of this study is to evaluate and analyse Italian physicians' knowledge and information level about the scientific and therapeutic potential of stem cells and to verify their inclination between embryonic and cordonal stem cell research. Despite their difference from hESC, cordonal stem cells are considered to be a valid therapeutic alternative. Moreover, the aim of this study is to evaluate and analyse physicians' attitudes towards the scientific and therapeutic utilization of stem cells and finally to verify which type of correlation exists between the current Italian legislation and the physicians' opinions. Regardless of the complexity of ethical implications, the survey revealed a good understanding of the benefits of stem cells research by the physicians. Ethical and juridical implications of stem cells utilization significantly depend on their source (skin, blood, embryo, etc.). Every biological source involves different criticality levels and normative difficulty.

### 5.1. Embryo-Derived Stem Cells

Human embryo research still represents a particularly controversial and complex matter [11]. Currently, in Italy a proper embryo-oriented legislation is in force (Law 40/2004). According to this law, couples have the right to be informed about the health status of their embryos (article 14.5). Moreover, clinical and experimental research for therapeutic and diagnostic purposes is also permitted, albeit in conditions of protecting the health and development of the embryo (article 13.2).

However, every invasive treatment to the embryo not aimed to diagnostic and therapeutic purposes is prohibited. The balance between the freedom of science and the embryo's health protection is settled in favour of the embryo. Our survey shows that the current legislation appears consistent with the opinion of 45% physicians interviewed (who did not favour human embryo research). Thus we can assume that the embryos come first, even when faced with the advancements of research.

The relatively high percentage of physicians in favour of embryos' use (34% and 44% for supernumerary embryos' use) demonstrates that several physicians believe in the potential of embryonic stem cells research, approving the embryo's sacrifice for protecting public health. The increased percentage (44%) in favor of using spare embryos could be justified by the minor disvalue that is generally attributed to the sacrifice of nonimplantable embryos. In contrast, the high percentage of abstentions (21% for embryos and 16% in case of supernumerary embryos) suggests that a still consistent fraction of the population of physicians lacks the perception of the importance of these issues and has little information and awareness about the benefits of the use of human embryos for research purposes.

The last decade of Italian debate about the embryo's condition and health has been changing.

At the beginning, Dulbecco's Commission provided a very open opinion on the donation of supernumerary embryos (Principle of beneficence) [12]. The National Bioethics Committee, instead, often underlined the necessity of a research not oriented to the destruction of the embryo [13].

Furthermore, in 2007, the above committee performed a study regarding the destiny of the embryos coming from ART and not implantable, recalling the need for determining criteria for the ascertainment of the embryo's death, in order to make the donation of stem cells for scientific purposes possible [14].

According to European legislation, the dispute between the embryos' production and utilization has been resolved by article 18, first paragraph, of the Convention on Human Rights and Biomedicine (1997), which prohibits the creation of human embryos for research purposes (art. 18, 1st comma, provides that “Where the law allows research on embryos in vitro, it shall ensure adequate protection of the embryo”) [15–17]. Such a rule continues stating, in the second paragraph, that “The creation of human embryos for research purposes is prohibited.” The use of human embryos in research does not appear prejudicially prohibited: the Convention on Human Rights and Biomedicine, in fact, simply leaves at the discretion of the single member States the opportunity to allow or not the use of embryos, not stating a clear and adequate level of protection.

According to a comparative approach, we can classify juridical models on embryos research into two main types: “value oriented,” characterized by very restricted rules aimed to guarantee the embryo preservation [18], and “procedure oriented,” where instead less restricted rules are in force which allow exceptions [19, 20]. While different countries present differences and similarities under many points of view (creation of human embryos/embryonic stem-derived cells lines, import of stem cell lines, etc.), almost all of them are characterized by a common condition: the lack of specific regulation on the right of the couple to be informed about the possibilities offered by the medical sciences in the field of embryo research. The major part of the juridical models examined, in fact, do not provide a specific policy aimed to promote the knowledge and information about the therapeutic and research potentialities of stem cells.

Some countries opted for very strict embryo's protection legislation, prohibiting every treatment not aimed at diagnostic and therapeutic purposes according to the first model (e.g., Austria and Poland) [21]. As for Poland, it is interesting to underline that the legal system does not have a specific regulation on the embryo and embryonic stem cells research, but the existing rules address the issue according to the principle that the life of the unborn child is protected from the conception. Furthermore, article 38 of the Polish Constitution ensures expressly the legal protection of the life of every human being [22]. By providing a more comprehensive regulation, Austria law bans research on embryos, including the derivation of embryonic stem cell lines, but similarly to Italy it does not present a specific regulation on the use of imported stem cells lines [23]. Other countries, according to the second model, allow certain technologies and treatments on the human embryo, after a strict monitoring and guideline respect (FR and DE, e.g., allow research on supernumerary embryos, within certain conditions, but prohibit the creation of human embryos for research purposes and for therapeutic cloning—France law no. 2013-715, Germany law no. 28 June 2002, and Portugal law no. 32/2006) [24–27]. Differently from Italy, Germany and France have a comprehensive regulation on the use of ES cells derived from IVG treatment though with different level of protection towards the human embryo. The German law, for instance, allows research only on the imported embryonic stem cell lines derived from IVG treatments before May 1, 2007. This date was postponed in 2008 by a new amendment to the 2002 Stem Cell Act (Stammzellgesetz) that was introduced thanks to the pressure of the scientific community. A more open approach was adopted very recently by the French legislator, which issued in 2013 an amendment (law no. 2013-715) to the previous law no. 2011-814, aimed to ease the existing regulation on the scientific research involving embryos and embryonic stem cells lines [28]. The new law abrogated the explicit ban regarding the embryo and embryonic stem cells research, allowing it under certain conditions (e.g., scientific impact, medical aim, and compliance with ethical principles). The employment of ES cells from supernumerary embryo derived from IVG treatment is allowed under the Portuguese law, even if the creation of human embryo for research purposes is prohibited.

Very liberal models occur in countries such as Spain, UK, Belgium, Sweden, Czech Republic, Greece, and Switzerland where the creation/use of embryos is allowed for research purposes under strict conditions, on the basis of the authorization and the monitoring of technical bodies. Clearly, UK represents one of the most liberal existing models in the European area, in which the Human Fertilisation and Embryology Act, dated on 1990—as modified by the further Human Fertilisation and Embryology Act (2001)—allows the use of embryos for research purposes as well as the therapeutic cloning [29–31]. Similarly to UK, in Belgium (Research on Embryos in vitro Act 2003) and Sweden (Activities Involving Human Eggs for Research or Treatment Purposes Act 1991), the use of embryos is allowed up to 14 days after the fertilisation [32, 33]. However, as for Belgian model, the general rule is that the creation of embryos exclusively for research purposes is prohibited, even if it is noteworthy to underline that this rule can be derogated according to the needs of the scientists. Both Greece (Law 3089/2002) and Czech Republic (Act on Research on Human Embryonic Stem Cells and Related Activities 2006) allow research on supernumerary embryos and/or on embryo-derived stem cells, but while the former consents the use of embryos up to 14 days, the latter limits the scientific use to 7 days after fertilization. Reproductive cloning is banned in both the countries mentioned above [34, 35]. Similarly to Greece, Switzerland allows research on supernumerary embryos up to 7 days after fertilisation as well as on imported embryonic stem cells (The Federal Act on Research Involving Embryonic Stem Cells 2003) [36].

In our survey, the high percentage of abstentions, 21% for embryos and 16% in case of supernumerary embryos, suggests the existence of a widespread detachment from some issues and also of misinformation and lack of awareness about the benefits of hESC based research.

The opportunity and the lawfulness of human embryos utilization for research purposes must be evaluated not only with regard to ethical principles but also according to a solidarity and collective convenience [37]. To the question whether human embryos health can prevail upon scientific freedom and research protection, the only answer is an impartial balance of the health interests involved for both the individual and the community. In order not to deprive the community of potential new discoveries in biomedical field, it might be necessary to establish the limit for embryos' protection.

However, at international level we notice, as already highlighted, a progressive opening towards supernumerary embryos research. The Convention on Human Rights and Biomedicine leaves in fact wide freedom to member States in order to decide about allowing hESC utilization underlining, at the same time, the importance and necessity of guaranteeing an adequate protection of embryos' health. Recently, the Court of Justice of the European Union also confirmed the necessity to avoid embryos destruction. In a judgment, the Court established that drugs arising from research on hESC cannot be patented [38, 39].

### 5.2. Umbilical Cord and Adult Stem Cells

By an ethical point of view, our survey confirms that the use of adult stem cells is difficult. Most of the physicians interviewed (87%) opted for cord blood cells rather than embryonic stem cells supply. Furthermore, most of them (73%) are in favour of autologous conservation considering its usefulness for therapeutic purpose, while only 16% are against it and 11% have no opinion on this issue. These results highlight that physicians trust the high benefits of umbilical cord stem cells and, at the same time, show how the current juridical frame (based on a greater protection of heterologous explant for solidarity purposes) does not appear to reflect the physicians' position. Physicians are consistently in favour of autologous explant considering it useful in order to safeguard their child's health.

However, as mentioned above, the Italian legislation on umbilical cord stem cells use is very strict, allowing the autologous storage only in peremptorily predetermined cases. After all, in Italy, the debate on adult stem cells storage is focused on two main issues: the “health protection”—the utilization of stem cells must represent a therapeutic protection for the individual use of the donor—and the “solidarity principle”—the use of stem cells must be a therapeutic protection at the whole community's disposal.

The resolution of the main juridical issues on stem cells research, both adults and embryonic, involves the requirement of balancing several interests issued from rights and freedoms protected by the Italian Constitution such as the right of health (art. 32 Const.), science freedom (art. 33 Const.), the promotion of scientific research (art. 9 Const.), and the freedom of economic initiative (art. 41 Const.). If on one hand the utilization of human cells could represent a solidarity act, on the other hand it could bring, to the exploitation of the human being for economic purposes, despising human dignity.

The current survey finally shows that the scientific misinformation and the influence of certain religious orientations affect the physicians' decision-making process. The lack of information does not concern only the scientific-therapeutic aspects but also the juridical ones: forty-nine % of physicians, in fact, appear to be misinformed about the Italian law on this matter. The lack of a suitable knowledge underlines not only the need for a wider diffusion of scientific information on this topic (e.g., through public initiatives aimed to create awareness in health care providers) but also the need for harmonizing Italian legislation with the other countries. Finally, participants' responses might be influenced by their religious orientation (most of the physicians involved were Catholic (56%)) since, in the scenario that animates the current Italian debate on these matters, the position held with particular emphasis by the exponents of the Catholic ethics appears stronger even with respect to other Catholic countries, like Spain, which have adopted an intermediate public policy on hESC and cloning research [40].

Eighty-seven % of the physicians interviewed, before a concrete alternative, gave their preference to umbilical cord stem cells rather than to hESC. However, when we asked the physicians about the opportunity to use human embryos for isolation of hESC without a concrete alternative, we registered an increase in the percentage of favourable answers (34% for embryos and 44% for supernumerary embryos). The current study suggests that today the religious influence on the physicians' decision-making process is not based any more on an* ab origine* refusal of human embryo research, but it can be converted into the need for a more embryo's protection-oriented research. Furthermore, since 2006, a Eurobarometer survey has been bringing out the fact that in countries with a high incidence of Catholic and Protestant belief (e.g., Italy and England), most of the physicians were in favour of human embryo research only in the presence of a more restricted legislation [41].

## 6. Conclusions

Data reveal the importance of improving knowledge and information about the therapeutic and research potential of stem cells [42].

Information about cryopreservation of cordonal stem cells is especially critical in gynaecologists and paediatricians since they are the main stem informer from physicians to future mothers. Sadly, the Italian percentage of cryopreserved cordonal stem cells is particularly low.

Although an appropriate scientific and technical knowledge of the matter is still lacking, ethical and juridical implications represent the major obstacle to embryonic stem cells research interfering with health care professionals' decisions. This is mainly due to the Italian cultural background based on historical reasons and on specific religious orientations currently involving Italian physicians. These orientations have an impact on the current and future Italian juridical system [43]. Human biological samples are already used for research, therapeutic procedures, and personal/genetic information. The European Directive 2004/23/CE and the Regulation 1394/2007/CE give directions about the coexistence of free public and private stem cells treatment systems. Thus, citizens are encouraged to donate, not only for public use but also for private use. Our study underlines, in view of the European Union legislation, the importance of an Italian shift toward European standards; this is also required in order to preserve personal freedom and the human right to dispose freely of one's own body parts as expressed in the Oviedo Convention (art. 22) and in the additional protocol to the Convention on Human Rights and Biomedicine, concerning biomedical research (art. 14).

In conclusion, we believe that human beings have the right to be informed in order to take decisions concerning their body parts, their biological tissues collection, and their relative destination and utilization [30].

## Figures and Tables

**Table 1:** Participants' general information.

Participants	Age (years)	Sex (%)	Religion (%)
Respondent (377 : 3.361—11%)	30–70	M (48.2)	Catholic (56)
		F (51.8)	Other (44)
Nonrespondent (2.984 : 3.361—89%)	30–65	M (68.8)	Catholic (71)
		F (31.2)	Other (29)

**Table 2:** Participants' medical specialties.

Respondents		Nonrespondents	
Gynaecology and obstetrics	(43%)	Hygiene	(0%)
Paediatrics	(41%)	Pathology	(0%)
Psychiatry	(1%)	Minor surgery	(0%)
Surgery	(1%)	Radiology	(0%)
Forensic medicine	(1%)	Geriatric medicine	(0%)
Dentistry	(1%)	Pathology	(0%)
Anaesthesia	(1%)	Ophthalmology	(0%)
		Orthopaedics	(0%)
		Cardiology	(0%)

**Table 3:** The questionnaire.

Questions	Yes	No	Do not know
(1) Do you have a generic knowledge of stem cells?	92%	4%	4%
(2) Do you have a specific knowledge of stem cells?	31%	59%	10%
(3) Are you aware of the potential therapeutic applications with stem cells?	86%	7%	7%
(4) Did you ever attend vocational and training courses or meetings regarding stem cells?	29%	65%	6%
(5) Would you be interested in developing your knowledge about stem cells?	70%	15%	15%
(6) Are you aware of the possibility to isolate stem cells from embryos (with their sacrifice), human tissues, and umbilical cord?	92%	3%	5%
(7) Do you agree with human embryo research?	34%	45%	21%
(8) Do you agree with human supernumerary embryo research?	44%	40%	16%
(9) Would you prefer the explant of stem cells from umbilical cord rather than from embryos in case this option would be legal?	87%	3%	10%
(10) Did you personally receive information about the explant methods of umbilical cord stem cells?	47%	47%	6%
(11) Do you know the Italian legislation about the explant and conservation of umbilical cord stem cells?	51%	40%	9%
(12) Do you know the difference between autologous and heterologous conservation of umbilical cord stem cells?	54%	38%	8%
(13) Would you donate part of your child's umbilical cord stem cells for therapeutic and research purposes?	91%	2%	7%
(14) Would you conserve your child's umbilical cord stem cells for personal and therapeutic purposes?	73%	16%	11%

**Table 4:** Participants' religious attitudes.

Religion	%	*n*
Catholics	(56%)	210
Atheists (agnostics)	(9%)	35
Jews	(1%)	2
Other	(2%)	6
Unknown	(21%)	79
